# “Scratches” of a Surgeon

**Published:** 1882-04

**Authors:** 


"SCRATHCHES" OF A SURGEON. By *Wm. Tod Helmuth, M. D.* Pp. 120. Chicago: Wm. A. Chatterton & Co. 1879.

Prof. Helmuth is almost as well known for his skill in making pen-scratches as he is for his knife-scratches. Those who have not yet seen these bright "Scratches," we advise to lose no time in doing so.

